# Triadic signatures of global human mobility networks

**DOI:** 10.1371/journal.pone.0298876

**Published:** 2024-02-23

**Authors:** Rachata Muneepeerakul, Jeffrey C. Johnson, Michael J. Puma, Michael A. Zurek-Ost

**Affiliations:** 1 Department of Agricultural and Biological Engineering, University of Florida, Gainesville, FL, United States of America; 2 Department of Anthropology, University of Florida, Gainesville, FL, United States of America; 3 Columbia Climate School, Center for Climate Systems Research, Columbia University, New York, NY, United States of America; New York State Museum, UNITED STATES

## Abstract

Global refugee and migrant flows form complex networks with serious consequences for both sending and receiving countries as well as those in between. While several basic network properties of these networks have been documented, their finer structural character remains under-studied. One such structure is the triad significance profile (TSP). In this study, the TSPs of global refugee and migrant flow networks are assessed. Results indicate that the migrant flow network’s size and TSP remain stable over the years; its TSP shares patterns with social networks such as trade networks. In contrast, the refugee network has been more dynamic and structurally unstable; its TSP shares patterns with networks in the information-processing superfamily, which includes many biological networks. Our findings demonstrate commonality between migrant and social networks as well as between refugee and biological networks, pointing to possible interdisciplinary collaboration—e.g., application of biological network theories to refugee network dynamics—, potentially furthering theoretical development with respect to both network theory and theories on human mobility.

## Introduction

Refugee and migrant movements represent the most fundamental [1] and most extreme reactions to natural and human-induced change. When viewed together, these movements form complex networks—with nodes representing countries and edges representing human movements among them—to which network analyses can be applied to gain insights into their structure and dynamics. For example, Davis et al. [2] computed a number of basic network properties of the global migrant flow networks for the period 1960 to 2000 and found increasing transitivity of the network, declining average path length, and a shift towards larger values for degree distributions, noting that these trends “strengthened the ‘small-world’ behavior.” More recently, Abel et al. [3] examined the structure of international migration networks, observing that these networks exhibit stability while having numerous short-term fluctuations over time.

While these efforts above have provided important insights into the basic network properties of the global refugee and migrant flow networks, they are based on low-order properties derived from pairwise relationships. Recent studies on complex networks have indicated that low-order properties alone provide an incomplete picture and highlighted the importance of higher-order network patterns [4–7]. As Lotito et al. [8] put it, “growing empirical evidence is now suggesting that a large number of such interactions are not limited to pairs, but rather occur in larger groups.” Therefore, to obtain deeper understanding into human mobility networks, the higher-order structures of these networks need to be examined. One such structure is the “triad significance profile” (TSP) [9], a pattern based on triads or three-node subgraphs.

To be sure, some triadic structure has been studied for human mobility networks, but the focus was on commuting patterns at a city or state level and at a daily timescale (see, e.g., Refs [10, 11]). Here, we focus on more permanent movements (refugees and migrants) at the global scale on an annual timescale or longer. By addressing the subject matter at different spatial and temporal scales, our work complements the existing body of knowledge. Additionally, focusing on the TSP—rather the patterns and dynamics of individual triads—has another unique benefit: complex networks can be grouped based on their TSPs into “superfamilies” [9]. When two networks are part of the same superfamily, a similarity in their structures is implied: a sign of shared underlying dynamics. When this happens for two networks from different fields, it hints at the potential for cross-pollination of the concepts and analytical methods in those fields. This potential has not been explored nor realized for human mobility networks and will be addressed in this paper.

In particular, we asked the following questions: What are the TSPs of the refugee and migrant flow networks? To which superfamily, if any, do they belong? What are the different mechanisms that give rise to these TSPs? The answers to these questions will lead to a deeper understanding and more holistic picture of human mobility networks as well as identify potential connections with networks in other fields, which would in turn suggest interdisciplinary linkages that will facilitate further theoretical development with respect to both network theory and theories on human mobility.

## Materials and methods

### Defining the networks

In this study, we analyzed two types of global human mobility networks: migrant-flow and refugee-flow networks. In these networks, the nodes represent countries, and the edges represent international movements of people (migrants or refugees). A directed edge from country A to country B was formed when there was a movement of people (i.e., flow) from country A to country B; the weight of the edge was the number of people who moved. These were *directed, weighted* graphs. For our main analysis—triad significance profile—with its focus on *topological* structure, a threshold was applied to convert them into *directed, unweighted* graphs. Here it is important to make clear the following distinction: in the migration research literature, the term “migration network” sometimes refers to a web of interpersonal relationships (e.g., friendship networks), which often are *undirected, unweighted* graphs—these are distinctly different from the global human mobility networks considered here. The following sections provide information on the data used to construct the global human mobility networks; trends in their lower-order network properties; and the steps involved in the triad significance profile analysis.

### Data

#### Migrant flows

Migrant stock data from the United Nations Department of Economic and Social Affairs [12] were used to estimate international flows via a demographic accounting, pseudo-Bayesian average of migration transitions and stayers with a closed accounting system [13]. Estimates for this population were first made available at https://doi.org/10.6084/m9.figshare.c.4470464 and later compared against other estimation approaches [14].

This study adopted version 9 of these estimates to examine the expanded time-period, spanning six time points between 1990 and 2020. It is important to note that the dataset includes both (non-refugee) migrants and refugees, with the number of non-refugee migrants being significantly higher than that of refugees. Consequently, a direct comparison between refugee flows (discussed in detail below) and estimates of migrant-only flows is not feasible with the available data. For the purposes of this analysis, the terms “migrants” and “migrant flows” are used to refer to this dataset.

#### Refugee flows

Refugee flow data are directly available from the United Nations High Commissioner for Refugees (UNHCR) in the forced displacement flow dataset (https://www.unhcr.org/refugee-statistics/insights/explainers/forcibly-displaced-flow-data.html). These annual data span from 1962 to 2022 but are filtered to match the timespan of the migrant flow estimates from 1990 to 2020. Additional aggregation of these refugee flows to 5-year intervals allows for a more appropriate comparison with results from the migration flow data. Of note, flow magnitudes differ substantially between these two datasets, where discrepancies between flow calculations are attributed to varying estimation and/or data collection methodologies, which should be remembered when interpreting this study’s results.

### Human mobility flow volume and flow network topology

**Fig 1:**
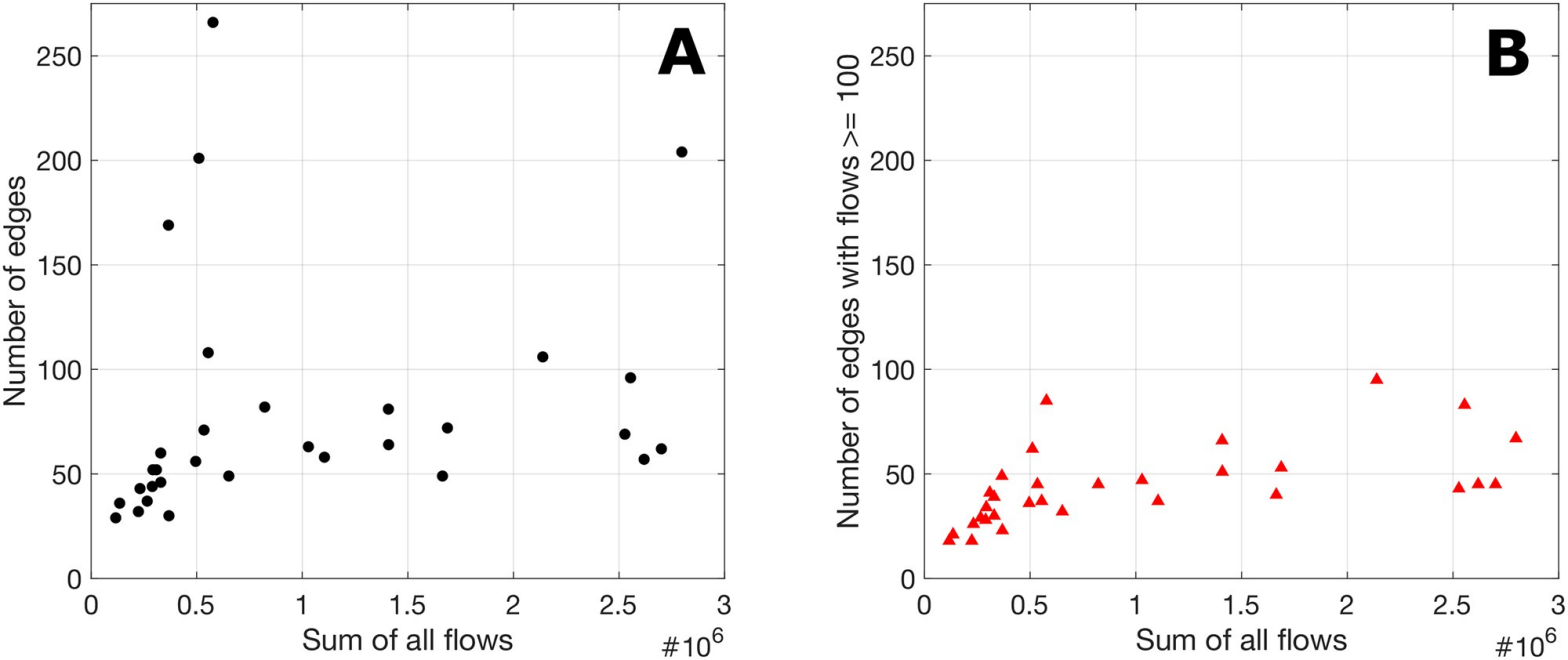
More flows, more edges? Not quite. The relationship between the sum of all refugee flows and the number of edges in the refugee flow network: (A) the original flow data, and (B) after removing edges with fewer than 100 refugees per year. The relationship is complex and exhibits significant variation, with certain years showing increased flows despite a reduced number of edges.

The total number of people moving around (i.e., volume) varies from year to year. It may be natural and intuitive to assume that years with more flows correspond with networks with more edges; however, that relationship is not so straightforward (Fig 1). A network can respond to changes in the total amount of flows by changing its number of edges (topological change) and/or changing the magnitude of its edges. The variation in the relationship between the sum of all refugee flows and the number of edges in the refugee flow network (Fig 1) reflects the variation in the degrees to which the human mobility network topologically responds to changes in human mobility flow volume. The focus on this study is on the *topology* of human mobility flow networks.

In particular, we focused on the topology of the global refugee and migrant flow networks consisting of edges with substantial flows. The rationale is that larger migration flows are more directly linked to societal impacts and have greater prospects for connecting with theories, while relatively low refugee flows have fewer impacts and may be caused by various idiosyncrasies that make connecting them to theories impossible. To that end, we chose the threshold of 100 people or more per year because it effectively removes the less significant edges, while preserving a dataset large enough for meaningful analysis. We applied the same threshold for the edges of both global refugee and migrant flow networks for consistency across our analyses. Using a constant threshold enables us to interpret more clearly the variation in the degrees to which the human mobility network responds to changes in human mobility flow volume (Fig 1). After applying the threshold, we treated these human mobility networks as unweighted, directed networks. It is also important to note that the unweighted networks capture the *diversity* of flows, while the weighted networks capture the *intensity* of flows, and that “both the diversity and the intensity of flows provide complementary, yet unique insights to understand migration systems”[5]. Here, the focus is on the *topological* structure (of the unweighted networks) and not on flow magnitudes.

### Triad significance profile

The higher-order network property of our interest is the triad significance profile (TSP). To determine the TSPs of the human mobility networks, we followed the method in Ref. [9], where Milo et al. used the network motif detection tool called ‘mfinder,’ available at www.weizmann.ac.il/mcb/UriAlon, to determine the TSPs of their various complex networks. The method is briefly described here for the paper’s self-containment; the interested reader is referred to Ref. [9] for further details. The main idea of this analysis is to compare the empirical frequency of each of the 13 triad types (shown at the bottom of Fig 5) to the frequency of the same triad type in a randomly rewired/shuffled network to determine the degree of over-representation (the empirical frequency is greater) and under-representation (the empirical frequency is less) of these triads. The pattern, or profile, of such over- and under-representation forms a TSP.

**Fig 2:**
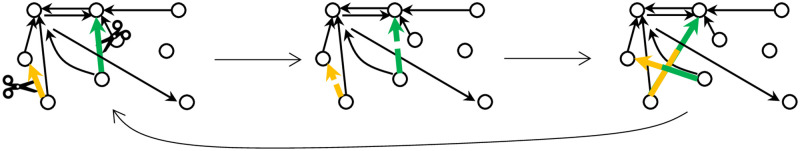
Shuffling for significance. Schematic diagram of the rewiring process to create a randomized network.

The rewiring process is done in such a way that the degree sequence and the numbers of mutual and asymmetrical edges remain the same. Starting with the empirical network of interest, one finds a pair of edges of the same type, namely either asymmetrical (nonreciprocal) or mutual (reciprocal), cuts, then swaps them (see Fig 2). This cut-and-swap procedure is repeated thousands of times until the network is thoroughly rewired. It is important to emphasize that this rewiring method preserves the in-degree and out-degree of each node. Other shuffling methods exist and have been used to study triadic patterns (e.g., Ref. [4]) that maintain only the overall number of mutual and asymmetric edges in the network, but not at the node level. That is, the method used here preserves more lower-order properties at the node level, while randomizing any structure of higher-order (including triadic) properties.

For each network under consideration, we produced such 200 randomly rewired networks. For each rewired network, the numbers of 13 triad types are tallied. A score *Z*_*i*_ for each triad type *i* is calculated as follows *Z*_*i*_ = (*n*_*i*, *E*_ − 〈*n*_*i*, *R*_〉)/*σ*_*i*, *R*_, where *n*_*i*,*E*_ is the number of triad type *i* in the empirical network, 〈*n*_*i*, *R*_〉 is the mean number of triad type *i* averaged across the 200 rewired networks, and *σ*_*i*, *R*_ is the standard deviation across the 200 rewired networks. The score is then rescaled as follows: $z_{i}=Z_{i}/(\sum_{i=1}^{13}Z_{i}^{2}{)}^{1/2}.$ The pattern of over- and under-representation of *z*_*i*_ is the network’s triad significance profile (TSP).

Finally, when investigating a fragmented network (as is the case for refugee flow networks, as will be shown shortly), we only considered the TSP of its largest component. Theoretically, the focus on the largest component is warranted because it contains the majority of the nodes in the network and thus is likely to be representative of the network as a whole. Methodologically, doing so avoided swapping edges among different fragmented components, some of which consist of isolated nodes of simple dyads (with no triads) and also maintained methodological consistency with the original TSP analysis and enabled the comparison with the already established superfamilies of complex networks [9].

## Results and discussion

**Fig 3:**
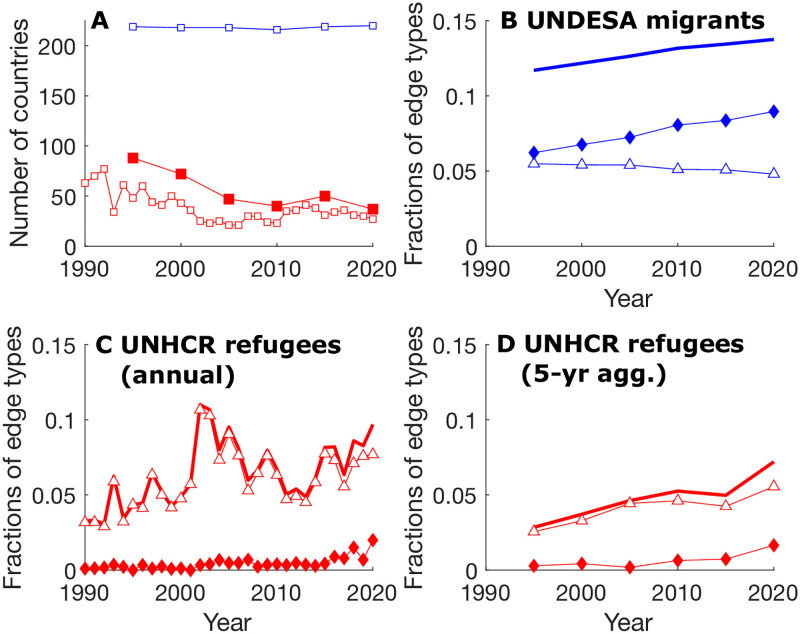
Trends of the low-order properties of the human mobility networks. (A) Numbers of countries involved with the human mobility networks composed of edges with 100 migrants/refugees or more per year; (B), (C), and (D) Edge compositions of the migrant, annual refugee, and 5-year aggregate refugee flow networks, respectively: diamonds = density of mutual edges (*m*), triangle = density of asymmetric edges *a*, and thick line = overall density (*m* + *a*). Here density is calculated as the fraction of the total number of possible *N*(*N* − 1)/2 node pairs, with *N* being the number of nodes, that are occupied by certain types of edges (mutual, asymmetric, or null).

**Fig 4:**
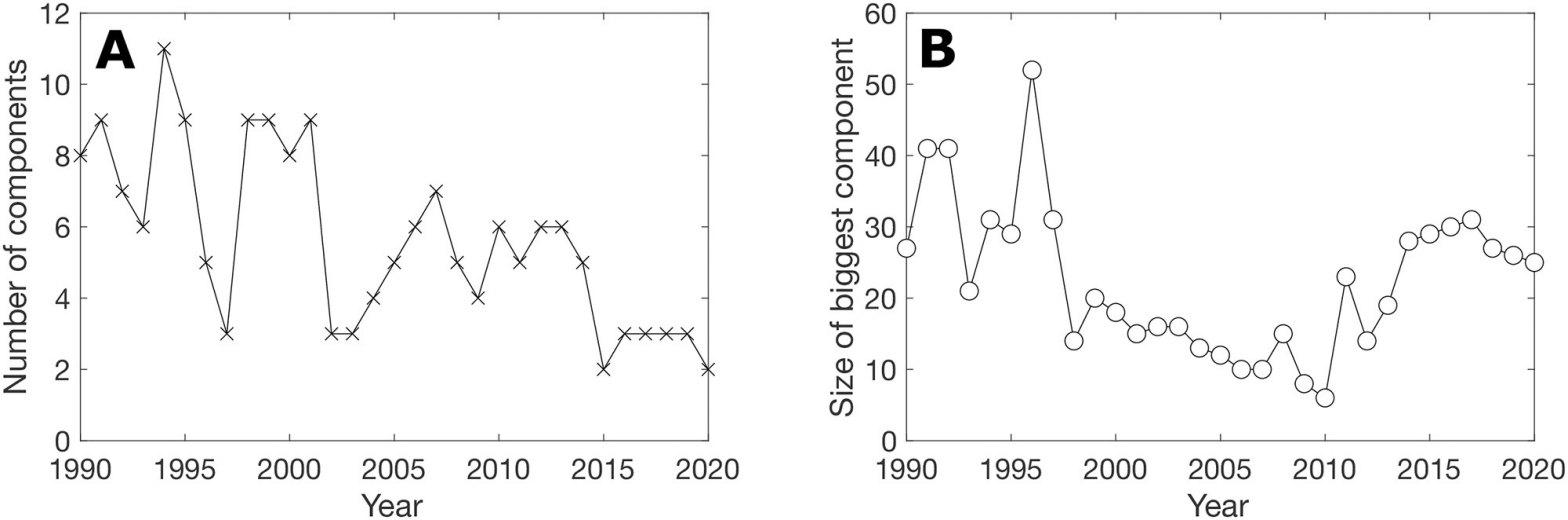
Fragmented nature of the refugee flow networks. (A) The numbers of disconnected components of the refugee flow networks across the years; and (B) The size (number of nodes) of the biggest component in each year.

**Fig 5:**
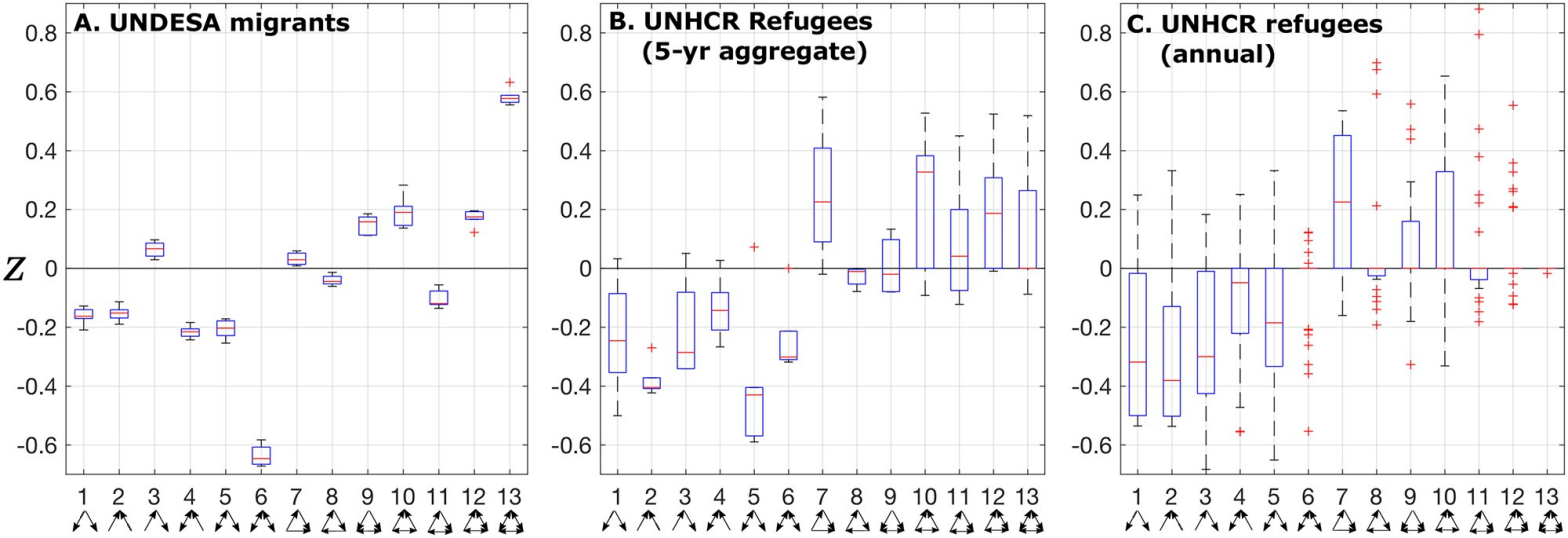
Triadic signatures of the human mobility networks. (A) UNDESA migrants; (B) 5-year aggregate UNHCR refugees (only largest components); and (C) annual UNHCR refugees (only largest components). For each box plot, central red mark = median; bottom edge = 25^th^ percentile; top edge = 75^th^ percentile; whiskers extend to the most extreme data points not considered outliers; red plus signs represent data points considered outliers [16].

Here, we first reported and discussed lower-order network properties, namely the network size, the overall density and the density of mutual and asymmetric edges (Fig 3), which, in addition to being informative in their own rights, impose constraints on the higher-order properties, including the TSP [4]; statistics of selected basic network properties are reported in S1–S3 Tables. We also considered the fragmented nature of the refugee flow networks—another distinguishing feature from the migrant flow network (Fig 4). We then reported and compared the TSPs of the UNDESA migrant flow network, the 5-year aggregate UNHCR refugee flow network (to match the timescale of the UNDESA data), and the original annual UNHCR refugee flow network (Fig 5).

### Trends of the low-order properties of the human mobility networks

Compared to the UNHCR refugee flow network (both annual and 5-year aggregate), the UNDESA migrant flow network is both larger in size and denser in its connections (Fig 3). The size of the UNHCR refugee flow network has been decreasing over time (Fig 3A). The density of mutual connections within the migrant flow network has increased over time, while the density of asymmetric edges has decreased (although the overall density has increased) (Fig 3B). Most edges in the UNHCR refugee flow network are asymmetric (Fig 3C and 3D). While becoming smaller in size, the UNHCR refugee flow network is becoming denser, with greater fluctuation at the annual time scale (Fig 3C and 3D).

### Fragmented nature of the refugee flow networks

The UNHCR refugee flow networks at both annual and 5-year aggregate scales—before and after applying the threshold—are fragmented (Fig 4). Over the last 30 years, the refugee flow network has become less fragmented—as indicated by the decreasing trend of its number of disconnected components (Fig 4A). This decline in fragmentation is partially due to the dominance of super-regional events driving the need for movement such as in Central, East, and West Africa and the Middle East. State-based, non-state based, and one-sided violence has been increasing during the period of 1989–2001 across these regions of Africa contributing to increased regional connectivity among countries and subsequently leading to a large single network component involving these African nations [15]. As discussed in the ‘triad significance profile’ section, when analyzing these refugee flow networks, we focused on the TSP of the largest component for a given year. Note that these largest components were always situated in Africa, with occasional connections to the Middle East in some periods.

### Migrants vs. refugees

The UNDESA data represent the cumulative change over the five-year periods between their reportings; to conduct a fair comparison between the UNDESA migrant and UNHCR refugee flow networks, we aggregated the UNHCR refugee flow data so that the aggregated flows also represent the cumulative changes over the same five-year periods (see Fig 5A and 5B). With the clear over-representation of type 13 (sometimes called “clique”—all three have reciprocated ties as in ‘a friend of a friend is also your friend’) and under-representation of type 6 (sometimes called “frustration”—my friend’s friend is my enemy), the migrant flow network fits well within the social network superfamily identified in Milo *et al.* [9] (Fig 5A). In other words, the migrant flow network has all the properties of normal human social networks such as friendship, exchange, advice networks, and small-world behavior, and more importantly the tendency for network closure. It is important to note that the social network superfamily in Ref. [9] was established based on the networks of individual friendships and of websites, while the migrant flow network operates at the global scale with countries as nodes: the fact that the networks of vastly different scales exhibit similar structure is indicative of some underlying scale-invariant governing dynamics.

More surprising is the structure of the other human mobility network—the refugee flow network. With the over-representation of types 7 and 10 along with the under-representation of types 1 through 6 (Fig 5B), the refugee flow network is closest to the information-processing network superfamily identified in Ref. [9], whose members include many biological networks such as neurons. This is the most surprising finding in this study. The fact that the refugee flow network exhibits similar network structure to biological networks—some of which operate at very small scales—again is indicative of some underlying scale-invariant governing dynamics.

In this case, an interesting place to start looking for such dynamics is the evolutionary optimization of networks [17]. In particular, Kaluza and colleagues showed that the TSP of the information-processing network superfamily corresponds to the optimal network structure for signal processing that is robust against link deletion [17]. In the context of refugee flow networks, input nodes represent sending countries and output nodes are receiving countries. This implies that if a pathway is blocked (link deletion), the network structure is such that refugees can find alternative pathways to reach desired destinations (signal processed). Also, Herbert and Ostojic [18] look at the lower order properties of neural networks in terms of connectivity and sparseness which appear strikingly similar to the properties and dynamics of refugee networks. These findings hint at interdisciplinary collaboration and for applying theories and computational and mathematical models of biological networks to further understand refugee network dynamics at the global scale.

At both 5-year aggregate and annual time scales, the refugee flow network exhibited greater fluctuations (wider ranges of the plots) than the migrant flow network. This difference is likely derived from the nature of their potential drivers. Key drivers of migration include economic opportunities and social ties (e.g., Ref. [19]). Changes in these drivers tend to be more slow-paced and predictable than natural disasters and violent conflicts that drive many refugee events.

At the annual scale, the refugee flow network shares some similarity in TSP patterns with its 5-year aggregate counterpart, e.g., over-representation of type 7 and under-representation of types 1 through 5, but with even greater variation (Fig 5C). Indeed, the greater variation—as well as the greater degree of fragmentation—made it more difficult to detect clear mechanisms behind, and contexts of, the TSP pattern of the refugee flow network. On the other hand, triads based on the 5-year aggregate data implied that these triads either involved very large amounts of refugee flows in some years or sustained substantial amounts of flows throughout a 5-year period; both factors made it easier to detect underlying mechanisms. As such, we focused on the 5-year aggregate refugee flow network in the following analysis.

### Drivers of the variation in the refugee flow network’s TSP

It is instructive to understand the relationships between these triad types in the context of refugee flows. For example, adding one directed edge to either triad type 1, 2, or 3 results in triad type 7. This suggests a situation in which refugees have certain destinations they wish to reach; if they cannot reach those destinations directly, they would find another way through a transit country. Similarly, it is instructive to note that types 10 and 11 can be obtained by adding a “return migration” tie to a type 7 triad: adding a return migration tie from the transit country to the origin in a type 7 triad turns it into a type 10; adding a return migration tie from the destination country to the origin in a type 7 triad turns it into a type 11. In the context of refugees, triad types with mutual edges (types 9 through 13) may be indicative of the “confused” situation and mindset: no one knows for sure where is safe, so they move back and forth.

**Fig 6:**
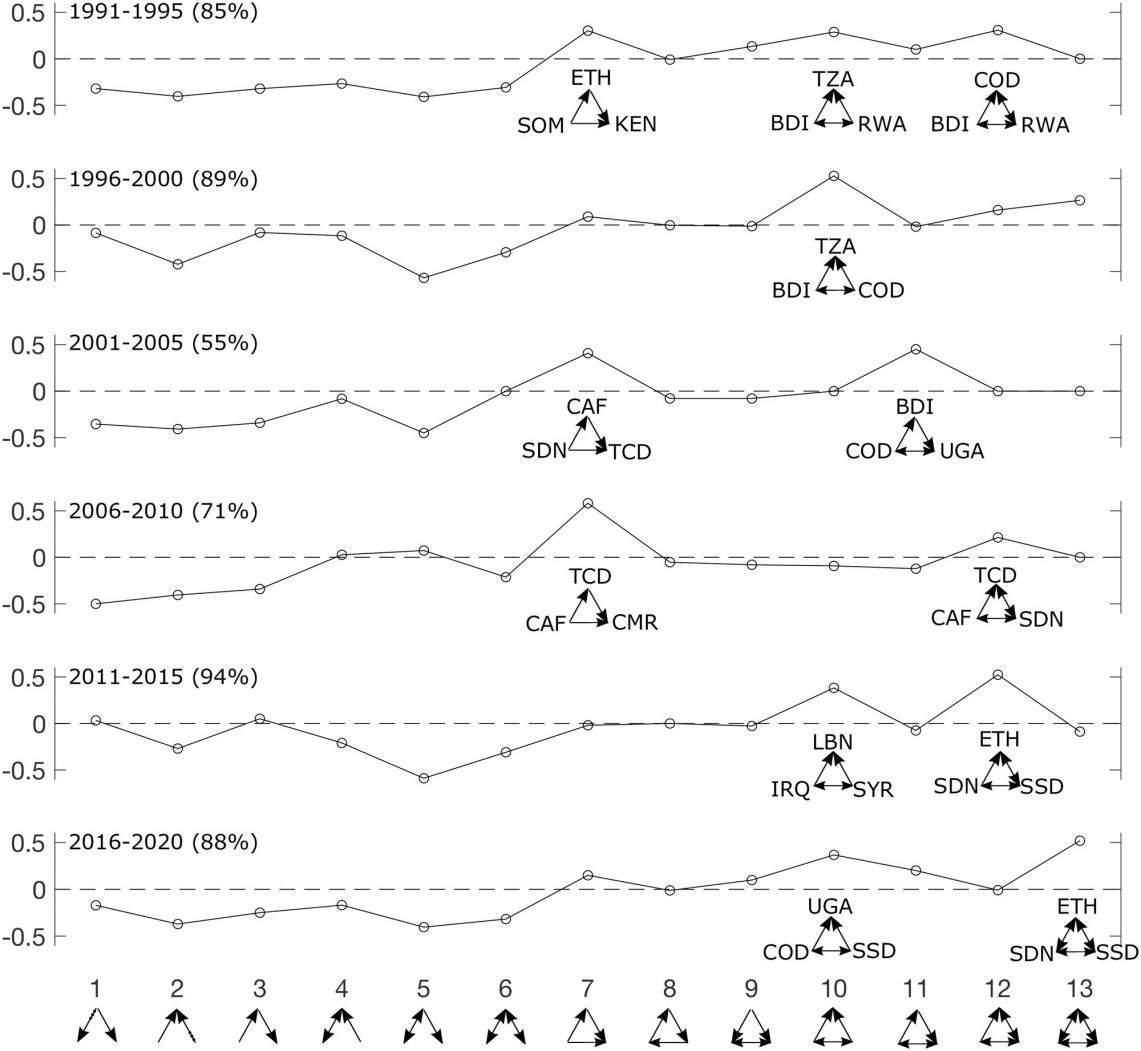
Example triads that shaped the TSP of the UNHCR refugee flow network. For each year, we considered triads of the strongly over-represented types; for each of these over-represented types, the triad with the greatest volume was shown. The percentage of total refugee flows contained in the largest network component (from which the TSP is derived) is shown in the parentheses: for the 2001–2005 and 2006–2010 periods where the percentages were relatively low compared to the other periods, the second biggest components contain 21% and 23% of total refugee flows, respectively—that is, the largest components still contain significantly more refugee flows. The country acronyms are defined as follows: BDI = Burundi; CAF = Central African Republic; COD = Democratic Republic of the Congo; CMR = Cameroon; ETH = Ethiopia; IRQ = Iraq; KEN = Kenya; LBN = Lebanon; RWA = Rwanda; SDN = Sudan; SOM = Somalia; SSD = South Sudan; SYR = Syria; TCD = Chad; TZA = Tanzania; UGA = Uganda.

To better understand the context under which the TSP of the refugee flow network has varied over the last 30 years, we considered specific triads that shaped the TSPs of the 5-year aggregate refugee flow networks (with the cutoff of 500 refugees or more, i.e., 100 refugees per year or more, applied to the edges) (Fig 6). In particular, we focused on the triads that belonged to the types that were strongly over-represented.

Many of these triads are made up of African countries (recall that the largest components of the refugee flow networks were situated in Africa) (Fig 6), reflecting specific regional phenomena possibly creating the opportunity for such triadic structures to emerge. Many of the over-representations of various triads in the refugee TSPs reflect regional conflicts often involving multiple contiguous countries. The most prominent of these over-represented triads are associated with what might be called “Congo’s cycle of rebellion.” The Democratic Republic of the Congo (COD) was recently involved in a series of conflicts, particularly from 1996 through 2012. Much of this conflict involved the COD neighbors of Burundi, Rwanda, and Uganda. For example, in the Congo war of 1998–2003 both Rwanda and Uganda backed different rebel groups fighting in the COD [20, 21]. Thus, refugees were driven across the various borders creating triadic movements, or “cycles,” during this period (Fig 6). Similarly, the over-represented triadic structures in later years (2005 forward) are another example of how regional conflicts seem to drive the emergence of these structures in refugee networks. Yet another example of regional conflicts and triadic emergence mostly involved Sudan and eventually the establishment of an independent South Sudan. The Second Sudan War “ended” in 2005 and was characterized by large loss of life and human misery. Concurrently, there was the Ethiopian occupation of Somalia in addition to other regional conflicts. Thus, the triadic structures in this period reflect the dynamics of regional conflicts in two specific geographic regions of Africa.

### Cross-scale connections: Future work

Finally, it is worth-reemphasizing the scales of our analysis: we focused on the patterns at the global spatial scale and annual or longer (5-year) time scale. Much can still be learned from the connections between these macro-scale patterns and those at smaller scales. As one moves toward smaller scales, a node’s representation changes from country to state to county to city or even a smaller unit. At these meso- and micro-scales, human mobility research also considers inter-personal relationship networks, where a node represents a person, not a place [22–24]. Connections can be made across temporal scales, too. As mentioned earlier, some research on triads (but not the TSP) has been conducted on human mobility at much shorter timescales, e.g., daily [10, 11]. In linking patterns across these time scales, more recent methods specifically designed for motifs in temporal networks (see, e.g., Refs [25, 26]) hold exciting promise moving forward.

In addition to the patterns of the mobility networks themselves, their relationships with other potential drivers change as one moves across spatial and temporal scales. Determining such relationships between network data requires methods that properly treat the interdependence typical in network data (see, e.g., Refs [27–29]). Integrating these diverse methods and applying them to human mobility networks at different spatial and temporal scales opens up exciting research avenues. This multidisciplinary approach promises to advance theoretical development in network theory as well as our understanding of human mobility.

## Conclusion

In this study, we set out to address the following questions: What are the TSPs of the global refugee and migrant flow networks? To which superfamily, if any, do they belong? What are the different mechanisms that give rise to these TSPs? We found that, despite both representing global human mobility networks, the migrant and refugee flow networks exhibit different patterns and dynamics. The global migrant flow network shares its structure with trade and social networks, while remaining stable over time. Conversely, the TSPs of the global refugee flow network—more precisely, of its largest component—fluctuates considerably, and closely resemble the superfamily of information-process networks, whose members include several biological networks. The largest components of the refugee flow networks across the years were situated in Africa, and their TSPs seemed to be driven by regional conflicts. These findings imply that knowledge from social networks can potentially help us understand the migrant flow network better, and likewise, knowledge from biological networks for the refugee flow network, thereby pointing to the possibility of cross-pollination—e.g., biological network theories applied to the refugee flow networks—to potentially further theoretical development with respect to both network theory and theories on human mobility.

## Supporting information

S1 Table Statistics of selected graph-theoretic metrics of the UNHCR refugee flow networks. (DOCX)

S2 Table Statistics of basic graph-theoretic metrics of the 5-year aggregate UNHCR refugee flow networks. (DOCX)

S3 Table Statistics of basic graph-theoretic metrics of the UNDESA migrant flow networks. (DOCX)
