# Supplementary material for: Triadic signatures of global human mobility networks
Source: PLoS One. 2024 Feb 23;19(2):e0298876. doi: 10.1371/journal.pone.0298876 (PMC10889869; doi:10.1371/journal.pone.0298876)
Supplement: S1 Table — (DOCX) [file pone.0298876.s001.docx]

**Table S1 Statistics of selected graph-theoretic metrics of the UNHCR refugee flow networks.** These networks were derived from applying the flow threshold of 100 people per year (see main text; they were directed and unweighted graphs. Asym stands for asymmetric (i.e., one-headed arrow); deg stands for degree. The minimum in-degree and minimum-out degree are zero for all years: in each year, there existed at least one country that was exclusively sending or receiving. Mean in-degree and mean out-degree are always equal and reported in the last column. Low values of degrees correspond to the fragmented nature of these networks (see main text).

| **Year** | **# nodes (countries)** | **Asym edges** | **Mutual edges** | **min in-deg** | **max in-deg** | **min out-deg** | **max out-deg** | **mean deg** |
| --- | --- | --- | --- | --- | --- | --- | --- | --- |
| 1990 | 63 | 62 | 2 | 0 | 5 | 0 | 8 | 0.45 |
| 1991 | 70 | 77 | 3 | 0 | 6 | 0 | 8 | 0.56 |
| 1992 | 77 | 85 | 5 | 0 | 4 | 0 | 11 | 0.65 |
| 1993 | 34 | 33 | 2 | 0 | 4 | 0 | 6 | 0.25 |
| 1994 | 61 | 59 | 4 | 0 | 4 | 0 | 8 | 0.46 |
| 1995 | 48 | 49 | 0 | 0 | 5 | 0 | 9 | 0.33 |
| 1996 | 60 | 73 | 6 | 0 | 9 | 0 | 8 | 0.58 |
| 1997 | 44 | 60 | 1 | 0 | 6 | 0 | 8 | 0.42 |
| 1998 | 41 | 41 | 2 | 0 | 3 | 0 | 6 | 0.31 |
| 1999 | 50 | 51 | 1 | 0 | 4 | 0 | 8 | 0.36 |
| 2000 | 43 | 43 | 1 | 0 | 4 | 0 | 7 | 0.31 |
| 2001 | 36 | 36 | 0 | 0 | 4 | 0 | 4 | 0.24 |
| 2002 | 25 | 32 | 1 | 0 | 4 | 0 | 6 | 0.23 |
| 2003 | 23 | 26 | 1 | 0 | 3 | 0 | 5 | 0.19 |
| 2004 | 25 | 22 | 2 | 0 | 2 | 0 | 5 | 0.18 |
| 2005 | 21 | 19 | 1 | 0 | 5 | 0 | 4 | 0.14 |
| 2006 | 21 | 16 | 1 | 0 | 2 | 0 | 4 | 0.12 |
| 2007 | 30 | 23 | 3 | 0 | 3 | 0 | 4 | 0.20 |
| 2008 | 30 | 28 | 1 | 0 | 4 | 0 | 5 | 0.20 |
| 2009 | 24 | 21 | 1 | 0 | 3 | 0 | 5 | 0.16 |
| 2010 | 23 | 16 | 1 | 0 | 2 | 0 | 4 | 0.12 |
| 2011 | 35 | 28 | 2 | 0 | 4 | 0 | 7 | 0.22 |
| 2012 | 36 | 31 | 3 | 0 | 4 | 0 | 6 | 0.25 |
| 2013 | 41 | 37 | 3 | 0 | 4 | 0 | 10 | 0.29 |
| 2014 | 38 | 41 | 2 | 0 | 4 | 0 | 11 | 0.31 |
| 2015 | 31 | 36 | 2 | 0 | 5 | 0 | 7 | 0.27 |
| 2016 | 34 | 41 | 5 | 0 | 5 | 0 | 8 | 0.35 |
| 2017 | 36 | 35 | 5 | 0 | 4 | 0 | 7 | 0.31 |
| 2018 | 31 | 33 | 7 | 0 | 4 | 0 | 8 | 0.32 |
| 2019 | 30 | 33 | 3 | 0 | 5 | 0 | 6 | 0.27 |
| 2020 | 27 | 27 | 7 | 0 | 5 | 0 | 6 | 0.28 |
