# Supplementary material for: Triadic signatures of global human mobility networks
Source: PLoS One. 2024 Feb 23;19(2):e0298876. doi: 10.1371/journal.pone.0298876 (PMC10889869; doi:10.1371/journal.pone.0298876)
Supplement: S2 Table — (DOCX) [file pone.0298876.s002.docx]

**Table S2 Statistics of basic graph-theoretic metrics of the 5-year aggregate UNHCR refugee flow networks.** These networks were derived from applying the flow threshold of 500 people per period (100 people per year) (see main text); they were directed and unweighted graphs. Asym stands for asymmetric (i.e., one-headed arrow); deg stands for degree. The minimum in-degree and minimum-out degree are zero for all years: in each year, there existed at least one country that was exclusively sending or receiving. Mean in-degree and mean out-degree are always equal and reported in the last column. Low values of degrees correspond to the fragmented nature of these networks (see main text).

| **Period** | **# nodes (countries)** | **Asym. edges** | **Mutual edges** | **min in-deg** | **max in-deg** | **min out-deg** | **max out-deg** | **mean deg** |
| --- | --- | --- | --- | --- | --- | --- | --- | --- |
| 1991-1995 | 88 | 98 | 11 | 0 | 6 | 0 | 13 | 0.82 |
| 1996-2000 | 72 | 84 | 11 | 0 | 6 | 0 | 10 | 0.72 |
| 2001-2005 | 47 | 48 | 2 | 0 | 5 | 0 | 7 | 0.35 |
| 2006-2010 | 40 | 36 | 5 | 0 | 4 | 0 | 7 | 0.31 |
| 2011-2015 | 50 | 52 | 9 | 0 | 5 | 0 | 12 | 0.48 |
| 2016-2020 | 37 | 37 | 11 | 0 | 5 | 0 | 10 | 0.40 |
