# Supplementary material for: Triadic signatures of global human mobility networks
Source: PLoS One. 2024 Feb 23;19(2):e0298876. doi: 10.1371/journal.pone.0298876 (PMC10889869; doi:10.1371/journal.pone.0298876)
Supplement: S3 Table — (DOCX) [file pone.0298876.s003.docx]

**Table S3 Statistics of basic graph-theoretic metrics of the UNDESA migrant flow networks.** These networks were derived from applying the flow threshold of 500 people per period (100 people per year) (see main text); they were directed and unweighted graphs. Asym stands for asymmetric (i.e., one-headed arrow). The minimum in-degree and minimum-out degree are zero for all years: in each year, there existed at least one country that was exclusively sending or receiving. Mean in-degree and mean out-degree are always equal and reported in the last column. Low values of degrees correspond to the fragmented nature of these networks (see main text).

| **Reporting year** | **# nodes (countries)** | **Asym. edges** | **Mutual edges** | **min in-deg** | **max in-deg** | **min out-deg** | **max out-deg** | **mean deg** |
| --- | --- | --- | --- | --- | --- | --- | --- | --- |
| 1995 | 219 | 1309 | 1484 | 0 | 175 | 0 | 130 | 18.52 |
| 2000 | 218 | 1280 | 1599 | 0 | 172 | 0 | 142 | 19.39 |
| 2005 | 218 | 1277 | 1712 | 0 | 165 | 0 | 151 | 20.35 |
| 2010 | 216 | 1186 | 1872 | 0 | 166 | 0 | 154 | 21.34 |
| 2015 | 219 | 1213 | 1996 | 0 | 177 | 0 | 154 | 22.53 |
| 2020 | 220 | 1155 | 2158 | 0 | 175 | 0 | 160 | 23.68 |
